# Phylogenetic relationships and characterization of the complete mitochondrial genome of *Eriobotrya japonica* in southeast of China

**DOI:** 10.1080/23802359.2019.1692709

**Published:** 2019-12-18

**Authors:** Jun Yang, Nan Liu, Xue-Lian Zheng, Jin-Cheng Wu, Xu-Jian Lin, Guo-Hua Zheng

**Affiliations:** a College of Horticulture, Fujian Agriculture and Forestry University, Fuzhou, Fujian, China;; b College of Environmental and Biological Engineering, Putian University, Putian, China;; c Bureau of Agricultural Research of Jiaocheng District, Ningde City, Fujian, China

**Keywords:** *Eriobotrya japonica*, complete mitochondrial genome, phylogenetic analysis

## Abstract

*Eriobotrya japonica* is an evergreen fruit tree originating in southeastern China. Its fruit is juicy with a pleasant taste and considerable medical value. However, there is no complete mitochondrial (cmt) genome resource for this species. This is the first report of the cmt genome of *Eriobotrya japonica* from southeastern China. The whole cmt genome was 434,980 bp in size with 37.80% GC content. The cmt genome of *Eriobotrya japonica* contains 41 protein-coding genes, 22 tRNA genes, and 3 rRNA genes. A phylogenetic maximum-likelihood (ML) tree was constructed based on 22 mitochondrial genomes from plant species. *Eriobotrya japonica* grouped closely with other *Rosaceae* species, which provides strong support for the traditional classification.

*Eriobotrya japonica*, which belongs to the family *Rosaceae*, is one of the most important fruit crops in China (Chen et al. 2009). There are many germplasm resources of loquat in China and the plant is widely cultivated in China’s Fujian, Sichuan, and Zhejiang provinces. *Eriobotrya japonica* fruit is greatly appreciated for its medical value, unique flavor, and high concentration of essential nutrients such as minerals and carotenoids (Xu and Chen 2011). Because of its unusual harvest season, loquat fruit has a considerable economic advantage in the fresh market over other fruits (Besada et al. 2013). To date, studies on loquat have focused mainly on post-harvest physiology and fruit preservation (Pareek et al. 2014; Liu et al. 2019). Only a few studies have focused on its evolution. In the present study, the complete mitochondrial (cmt) genome of loquat was determined. We also explored its phylogenetic relationship with other plant species. This research may facilitate better understanding of the phylogenetic relationships among other species and the conservation genetics of *E. japonica*.

Young leaves of *E. japonica* were obtained from College of Horticulture, Fujian Agriculture and Forestry University in Fuzhou (Fujian, China, 119an, Chinarticulture) and were deposited Fujian Agriculture and Forestry University (No. FAFUYSJ01). Total genomic DNA was extracted using a modified version of the CTAB method (Bousquet et al. 1990). Then the obtained genomic DNA was used to construct an Illumina pair-end library. The library was sequenced using Illumina technology and yielded approximately 12.43 GB of raw data. After filtration using the FastQC software (Andrews 2014), High-quality clean reads of 12.38 GB were used to assemble the mt genome using SPAdes (http://bioinf.spbau.ru/spades) (Bankevich et al. 2012) and the cmt genome annotation was used with the online program GeSeq (Tillich et al. 2017). The annotated mitochondrial genome was submitted to the GenBank database under accession number MN481990.

The cmt genome of *E. japonica* was found to be 434,980 bp in length with 45.41% overall GC content. The nucleotide contents of A, T, G, and C in the mitochondrial genome were 27.28%, 27.29%, 22.55%, and 22.86%, respectively. A total of 71 unique genes were found in the mt genome, comprising 41 protein-coding genes, 22 tRNA genes, and rRNA genes. All the coding regions accounted for 17.64% of the whole genome.

In order to better elucidate the phylogenetic relationship between *E. japonica* and its relative species, mitochondrial genome sequences downloaded from the GenBank database of 22 species were used to construct phylogenic maximum-likelihood (ML) trees using RaxML software v 8.2.9 (Stamatakis 2014). The bootstrap values of ML trees were calculated using 1000 replicates. As shown in Figure 1, mitochondrial genome of *E. japonica* was most similar to that of the *Pyrus pyrifolia* and belonged to family *Rosaceae*. This is significant for further understanding of the phylogenetic evolution of the *E. japonica*.

**Figure 1.**
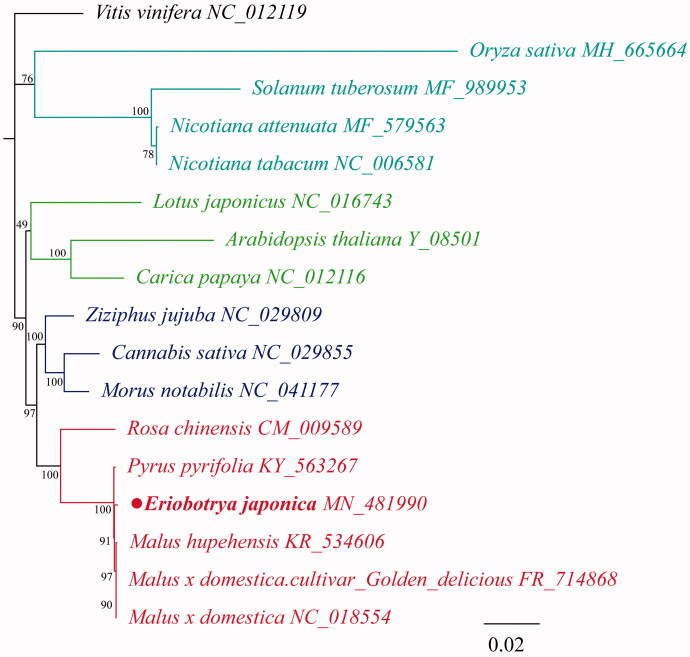
The maximum-likelihood (ML) phylogenetic tree was constructed based on of the complete plants mitochondrial genomes data of 17 species.
